# Efficacy, safety, and resistance profile of osimertinib in T790M mutation-positive non-small cell lung cancer in real-world practice

**DOI:** 10.1371/journal.pone.0210225

**Published:** 2019-01-09

**Authors:** Dong Kyu Oh, Won Jun Ji, Woo Sung Kim, Chang-Min Choi, Shin-Kyo Yoon, Jin Kyung Rho, Jae Cheol Lee

**Affiliations:** 1 Department of Pulmonary and Critical Care Medicine, Asan Medical Center, University of Ulsan College of Medicine, Seoul, Korea; 2 Department of Oncology, Asan Medical Center, University of Ulsan College of Medicine, Seoul, Korea; 3 Department of Convergence Medicine, Asan Medical Center, University of Ulsan College of Medicine, Seoul, Korea; Seoul National University College of Pharmacy, REPUBLIC OF KOREA

## Abstract

The efficacy and safety of osimertinib were demonstrated in clinical trials; however, real-world clinical data, particularly the resistance profile, are limited. Here, we investigated the efficacy, safety, and resistance profile of osimertinib in real-world practice. We reviewed medical records of T790M mutation-positive lung cancer patients who started osimertinib between February 2016 and June 2017. Molecular pathologic data of biopsy samples obtained after acquisition of resistance to osimertinib were also analyzed. The study included 23 patients with a median age of 59 years. The median follow-up duration was 11.9 months (IQR, 4.7–15.8). Objective response was achieved in 17 (73.9%) patients, and the disease was controlled in 22 (95.7%) patients. Median progression-free survival (PFS) was 7.4 months (95% CI, 3.6–11.0). Adverse events were minimal except for one case of pneumonitis. Of 14 patients experiencing disease progression, 10 underwent re-biopsy. The T790M mutation disappeared in seven patients (70%), and one showed wild-type conversion. PFS was shorter in the T790M-loss group than in the T790M-persistent group (4.4 vs. 7.7 months). Two patients with small cell transformation responded well to subsequent chemotherapy. One patient developed a C797S mutation that became undetectable after two cycles of gemcitabine and cisplatin followed by six cycles of pembrolizumab, after which the patient responded well to osimertinib. In conclusion, osimertinib showed favorable efficacy and safety in real-world practice comparable to those observed in clinical trials. Repeat biopsy after the acquisition of resistance to osimertinib is helpful to direct further treatment strategies.

## Introduction

Epidermal growth factor receptor (EGFR) mutations are reported in 11–43% of patients with non-small cell lung cancer (NSCLC), particularly in those with adenocarcinoma [1–5]. The most common mutations are exon 19 deletions and exon 21 L858R point mutation, which account for 35–69% and 21–48% of EGFR mutations, respectively [4–8]. NSCLC patients with these mutations show excellent responses to first- and second-generation EGFR-tyrosine kinase inhibitors (EGFR-TKIs) and an improved prognosis [9–11]. Although EGFR-TKIs are the standard first-line treatment for EGFR mutant lung cancer, the development of acquired resistance limits progression-free survival (PFS) to 10–12 months [12, 13].

Several mechanisms of resistance to first- and second-generation EGFR-TKIs have been identified in NSCLC [14–16]. The acquisition of the T790M mutation in the EGFR gene is the most common mechanism, accounting for 50–60% of resistance cases. The activation of parallel signaling pathways, including mesenchymal epithelial transition factor (MET), hepatocyte growth factor (HGF), and human epidermal growth factor receptor 2 (HER2) pathways, is observed in up to 20–30% of cases. Less than 1% of resistance cases involve histological transformation, such as epithelial to mesenchymal transition (EMT), and the BRAF V600E mutation and related downstream signaling. Although several drugs targeting these resistance mechanisms have been investigated [14, 15], the third-generation T790M mutant-selective EGFR-TKI osimertinib (TAGRISSO, AstraZeneca Pharmaceuticals) is currently the only drug approved by the Food and Drug Administration (FDA) besides dabrafenib (TAFINLAR, Novartis) plus trametinib (MEKINIST, Novartis) targeting the BRAF V600E mutation, which accounts for a small proportion of resistance cases [17–20].

Osimertinib demonstrated its efficacy and safety as second-line treatment for advanced NSCLC in a large, randomized, phase 3 clinical trial (AURA3 trial), which showed better PFS in the osimertinib group than in the pemetrexed and platinum-based doublet chemotherapy group (10.1 vs. 4.4 months) [21]. However, the use of this novel drug requires re-biopsy to determine the emergence of the T790M mutation. Considering the failure rate of re-biopsy, which can be as high as 30%, a substantial number of patients are not eligible for osimertinib treatment even when a successful biopsy detects the presence of the T790M mutation [22, 23]. This prompted the launch of another large, randomized, phase 3 clinical trial to compare osimertinib with first-generation EGFR-TKIs as first-line treatment for EGFR mutant lung cancer (FLAURA trial) [24]. In this trial, osimertinib showed superior efficacy over standard EGFR-TKIs in terms of PFS (17.2 vs. 8.5 months). Based on the results, the National Comprehensive Cancer Network (NCCN) guidelines included osimertinib as a first-line treatment option, particularly in patients with EGFR mutant lung cancer [20]. Confirmatory data regarding the efficacy and safety of the drug in real-world practice are currently awaited.

Despite the initial impressive effects, resistance to osimertinib, which limits the efficacy of the drug, has been reported [25, 26]. To overcome the resistance to osimertinib, approaches similar to those used to overcome the resistance to first- and second-generation EGFR-TKIs need to be investigated, and the mechanisms by which cancer cells acquire resistance to osimertinib need to be elucidated. Because the drug has only been available in real-world practice for months, increasing data on osimertinib resistance should be published in the near future. Herein, we present our real-world experience with osimertinib and define its resistance profile. The results suggest that repeat biopsy after the acquisition of resistance to the drug is helpful to guide further treatment strategies.

## Materials and methods

### Study design and population

The study was performed at Asan Medical Center, a tertiary referral teaching hospital in Seoul, Korea. The data were retrospectively retrieved from patients with NSCLC who started osimertinib between February 2016 and June 2017. The inclusion criteria were as follows: a) patients with histologically confirmed locally advanced or metastatic NSCLC (stage IIIB or IV), b) those harboring a common activating EGFR mutation, such as exon 19 deletions or exon 21 L858R mutation on initial biopsy, c) those who experienced clinical and/or radiologic progression after treatment with at least one first- or second-generation EGFR-TKI according to the Response Evaluation Criteria in Solid Tumors version 1.1 (RECIST 1.1), d) those harboring the T790M mutation detected by re-biopsy at the time of disease progression, and e) those treated with osimertinib (80 mg/day) orally during the study period. There was no limitation regarding the number of prior EGFR-TKIs or systemic chemotherapies received. Since the objective was to investigate the efficacy, safety, and resistance profile of osimertinib in real-world practice, patients enrolled in any other clinical trials were excluded from the study. Patients who did not undergo follow-up computed tomography (CT) scans after the initiation of osimertinib were excluded.

The study was approved by the Institutional Review Board of Asan Medical Center with the approval number 2017–0950. Because of the retrospective nature of the study, the requirement for informed consent was waived.

### Molecular diagnosis of EGFR mutations

The molecular diagnosis of EGFR mutations was performed by direct sequencing and peptide nucleic acid (PNA) clamping-based real-time polymerase chain reaction (PCR) analysis of formalin-fixed paraffin-embedded (FFPE) tissues. Direct sequencing, which is the gold standard for EGFR mutation analysis, was used routinely until December 2015. Deoxyribonucleic acid (DNA) was extracted from FFPE tissues, amplified by PCR, and then analyzed by standard sequencing after purification. In December 2015, a commercially available and cost-effective method termed PNA clamping-based real-time PCR (PNAClamp EGFR Mutation Detection Kit, PANAGENE, Inc., Daejeon, Korea) was introduced and was thereafter performed routinely for the molecular diagnosis of EGFR mutations in our hospital. In PNA clamping-based real-time PCR, the PNA clamping probe selectively binds to the wild-type DNA and inhibits its amplification, whereas the mutant DNA is selectively enriched and becomes detectable by PCR. Details of the two molecular diagnostic methods are provided in our previous study [27].

### Outcomes

Clinical follow-up assessments including physical examinations, radiologic evaluations, and molecular pathologic analyses were performed. Because of the retrospective nature of the study, the CT scans were performed at various time intervals, with an average of 4–8 weeks between the scans. Treatment response was evaluated according to RECIST 1.1 by experienced investigators. PFS was defined as the time from the initiation of osimertinib treatment to disease progression, death from any cause, or the last follow-up date (February 28, 2018). Overall survival (OS) was defined as the time from the initiation of osimertinib treatment to death or the last follow-up date. The objective response rate (ORR) was defined as the percentage of patients showing partial or complete response. The disease control rate (DCR) was defined as the percentage of patients with stable disease, partial response, or complete response. In the present study, the most favorable response to osimertinib during the whole study period was used to assess both ORR and DCR. Adverse events were assessed using Common Terminology Criteria for Adverse Events version 5.0 (CTCAE 5.0). Re-biopsy was performed at the time of disease progression, and the collected tissues were processed for molecular pathologic analyses using the methods described previously.

### Statistical analysis

Data are presented as the median (interquartile range [IQR]) for continuous variables and number (%) for categorical variables. The Kaplan-Meier method was used for survival analyses, and comparisons were made using the log-rank test. P < 0.05 was considered statistically significant. Statistical analyses were performed using the Statistical Package for Social Science (SPSS, Chicago, IL, USA) version 22.0 for Windows.

## Results

### Baseline characteristics of patients treated with osimertinib in real-world practice

**Fig 1:**
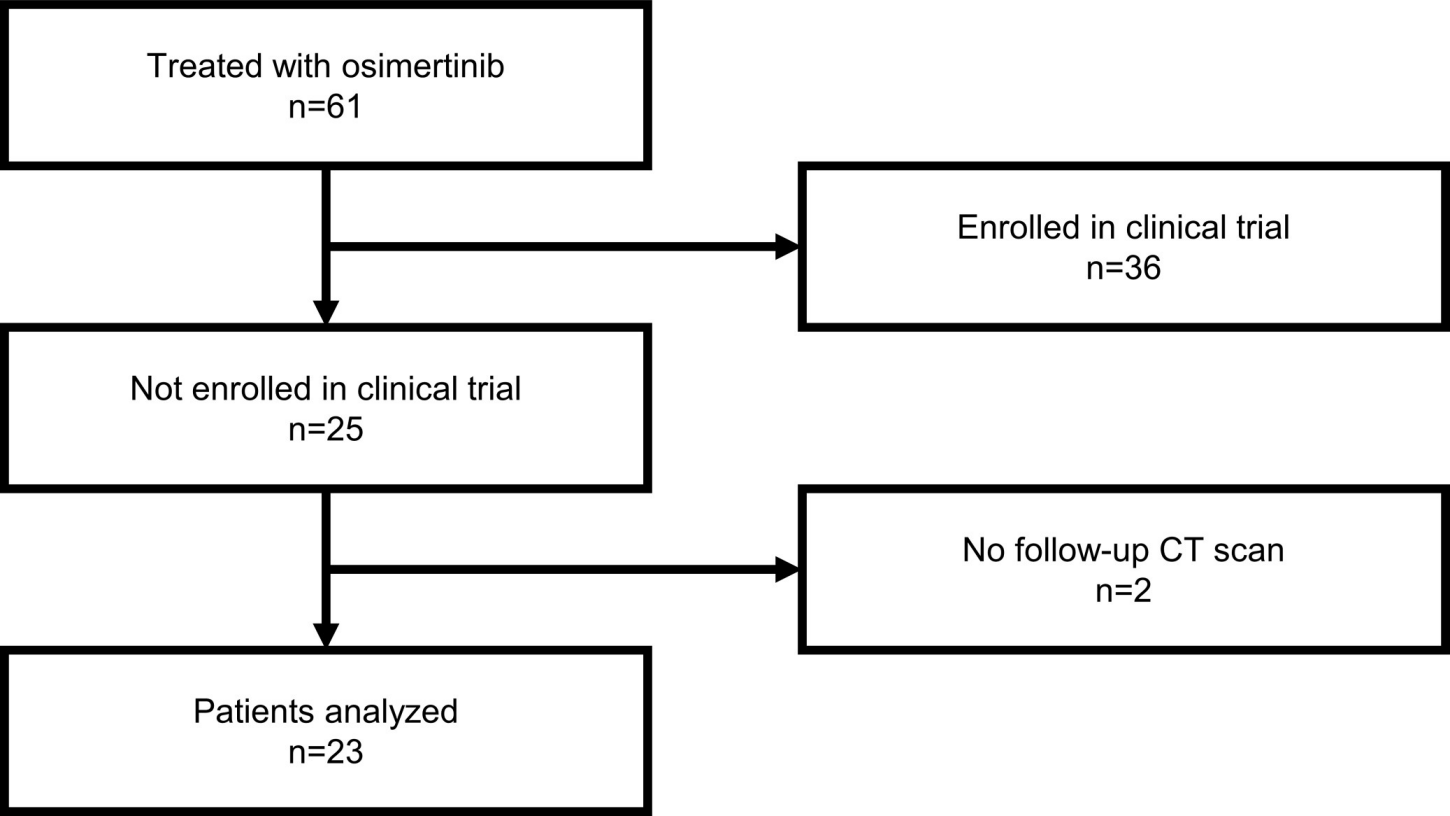
Flow chart of patient inclusion and exclusion.

During the study period, 61 patients started osimertinib treatment in our hospital. Among them, patients enrolled in other clinical trials (n = 36) and those who did not undergo follow-up CT scans (n = 2) were excluded from the study. Finally, 23 patients treated with osimertinib were included in the study (Fig 1). The reasons for not participating in the clinical trials were investigated as well. Of 23 patients, 16 (69.6%) refused to participate, 2 (8.7%) were not eligible because of recent exposure to other third-generation EGFR-TKIs such as olmutinib, and 5 (21.7%) patients were excluded from the clinical trials because of poor medical and/or performance status. Of these five patients, two (8.7%) had symptomatic central nervous systems (CNS) metastases, two (8.7%) had performance status grade ≥3, and one (4.3%) had concurrent breast cancer.

The baseline characteristics of the included patients are listed in Table 1. The median age was 59.0 years (IQR, 51.0–67.0), and there were 13 (56.5%) women. The median follow-up duration was 11.9 months (IQR, 4.7–15.8). All patients were diagnosed with adenocarcinoma on initial biopsy. All patients harbored the T790M mutation before the start of osimertinib treatment. Exon 19 deletions and exon 21 L858R mutation were present in 17 (73.9%) and 6 (26.1%) patients, respectively. The most common TKI received prior to osimertinib was gefitinib (n = 19 [68.2%]) followed by erlotinib (n = 6 [26.1%]), and three of these patients switched between the drugs because of adverse events such as skin eruption. Two patients began olmutinib treatment after acquiring resistance to gefitinib, and the treatment was changed to osimertinib after a short period because of grade 2 skin eruption.

**Table 1:** Baseline characteristics of the included patients (n = 23).

Variables	Number (%)
Age, years	
Median (IQR)	59.0 (51.0–67.0)
Gender	
Male	10 (43.5)
Female	13 (56.5)
Ethnic origin	
Asian	23 (100.0)
Performance status	
0–1	17 (73.9)
2–4	6 (26.1)
Mutation	
EGFR T790M mutation	23 (100.0)
EGFR mutations co-occurring with T790M	
Exon 19 deletion	17 (73.9)
L858R	6 (26.1)
Metastasis	23 (100.0)
CNS metastasis	9 (39.1)
Extra-thoracic metastasis	21 (91.3)
Previous treatments	
No. of previous systemic treatments	
1	9 (39.1)
2	7 (30.4)
≥3	7 (30.4)
No. of previous TKIs	
1	18 (78.3)
2	5 (21.7)
Previous TKIs	
Gefitinib	14 (60.9)
Erlotinib	3 (13.0)
Afatinib	1 (4.3)
Gefitinib, erlotinib	3 (13.0)
Gefitinib, olmutinib	2 (8.7)

Data are presented as number (%) or median (IQR).

Abbreviations: CNS, central nervous system; EGFR, epidermal growth factor receptor; IQR, interquartile range; TKI, tyrosine kinase inhibitor.

### Efficacy of osimertinib in real-world practice

**Fig 2:**
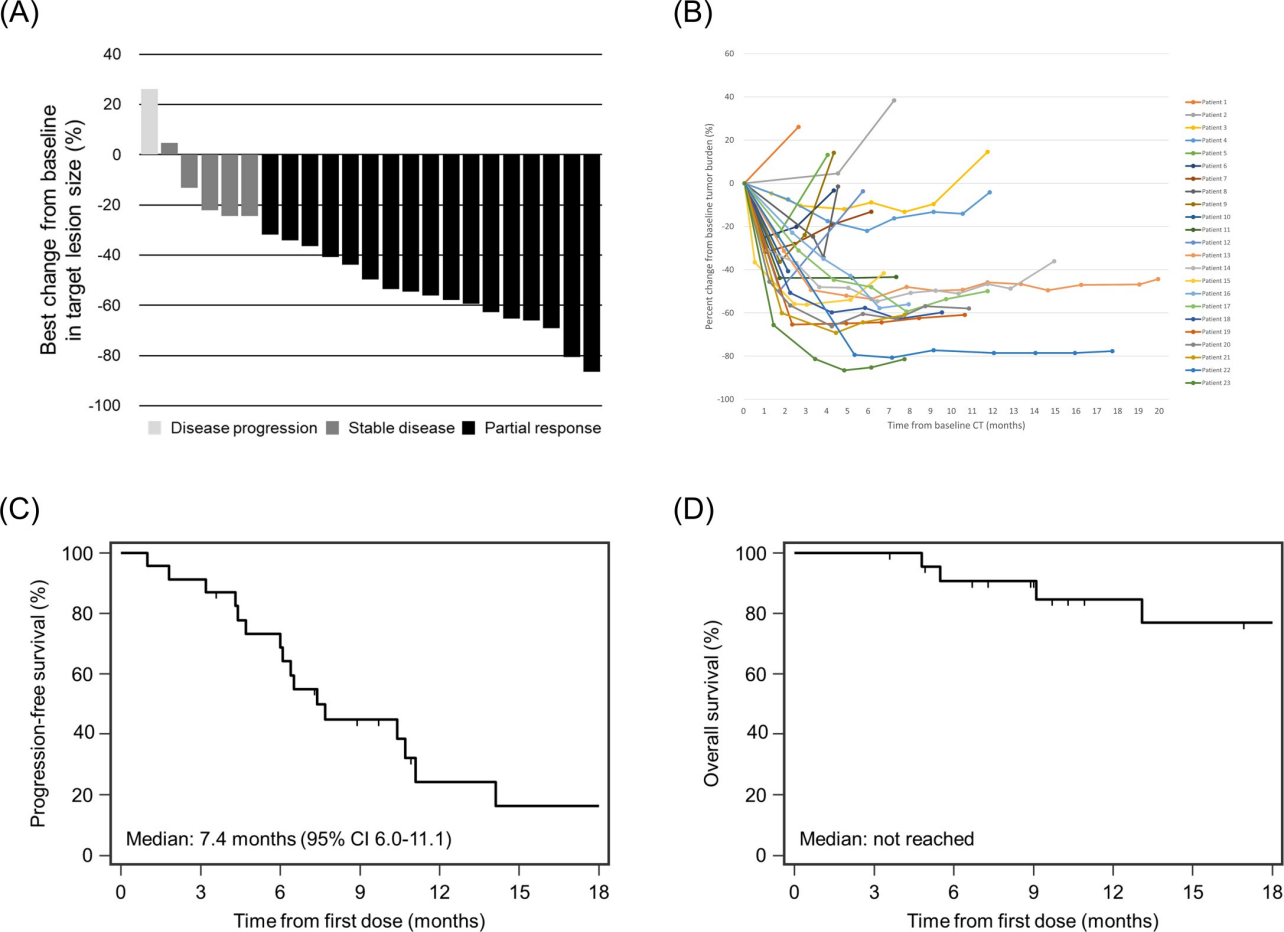
Response to osimertinib by RECIST 1.1 in real-world practice. (A) Waterfall plot of best percentage changes in target lesion size. (B) Spider web graph showing the percent change from baseline in the tumor burden. (C) Kaplan-Meier analysis of progression-free survival. (D) Kaplan-Meier analysis of overall survival. Abbreviations: CI, confidence interval; CT, computed tomography.

The median PFS was 7.4 months (IQR, 6.0–11.1), and the median OS was not reached (Fig 2). The proportions of patients estimated to be progression-free at 6, 9, and 12 months were 68.6%, 44.9%, and 24.1%, respectively. Although complete response to osimertinib was not achieved, objective response and disease control were achieved in 17 (73.9%; 95% CI, 53.5–87.5) and 22 (95.7%; 95% CI, 79.0–99.2) patients, respectively (Table 2). Of 17 patients showing an objective response, 10 (58.8%) had progressed or died at the time of the last follow-up.

**Table 2:** Response to osimertinib by RECIST 1.1 in real-world practice.

Variables	Number (%)
Complete response	0 (0.0)
Partial response	17 (73.9)
Stable disease	5 (21.7)
Disease progression	1 (4.3)
Objective response	17 (73.9; 95% CI, 53.5–87.5)
Disease control	22 (95.7; 95% CI, 79.0–99.2)

Data are presented as number (%).

Abbreviations: CI, confidence interval; RECIST, Response Evaluation Criteria in Solid Tumors.

### Adverse events related to osimertinib treatment in real-world practice

Twenty-two (95.7%) patients reported at least one adverse event, and 16 (69.6%) reported a potentially treatment-related adverse event as assessed by the investigators (S1 Table). The most common all-causality adverse event of grade ≥3 was anemia (n = 3 [13.0%]) followed by a decreased neutrophil count (n = 2 [8.7%]). Potentially treatment-related adverse events of grade ≥3 were minimal except for one case of pneumonitis, which improved with discontinuation of osimertinib and administration of corticosteroids.

### Resistance profile of osimertinib in real-world practice

**Fig 3:**
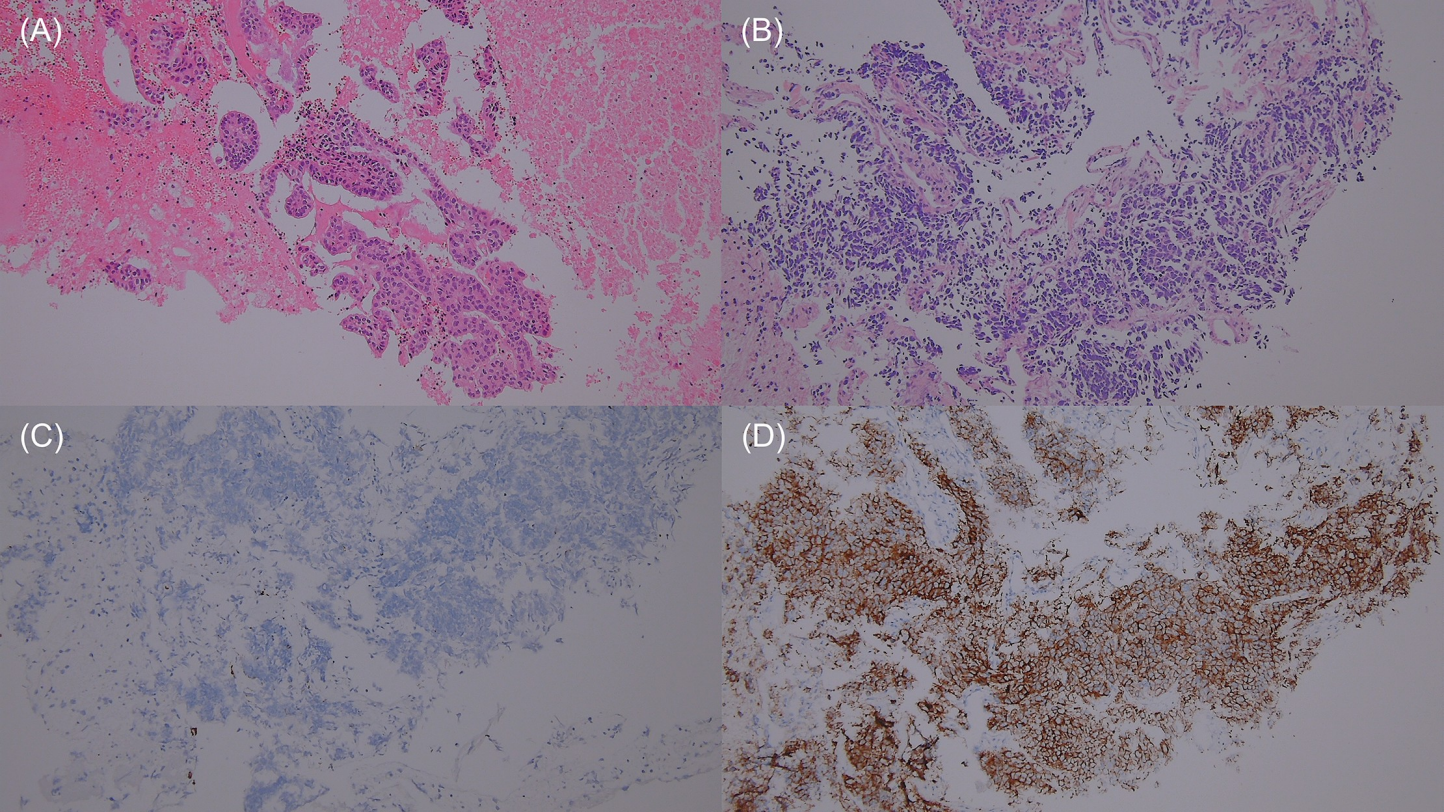
Transformation to small cell carcinoma from adenocarcinoma. (A) Adenocarcinoma, H&E; (B) small cell carcinoma, H&E; (C) small cell carcinoma, CK; (D) small cell carcinoma, CD56. Abbreviations: CD56, cluster of differentiation 56; CK, cytokeratin; H&E, hematoxylin and eosin.

**Fig 4:**
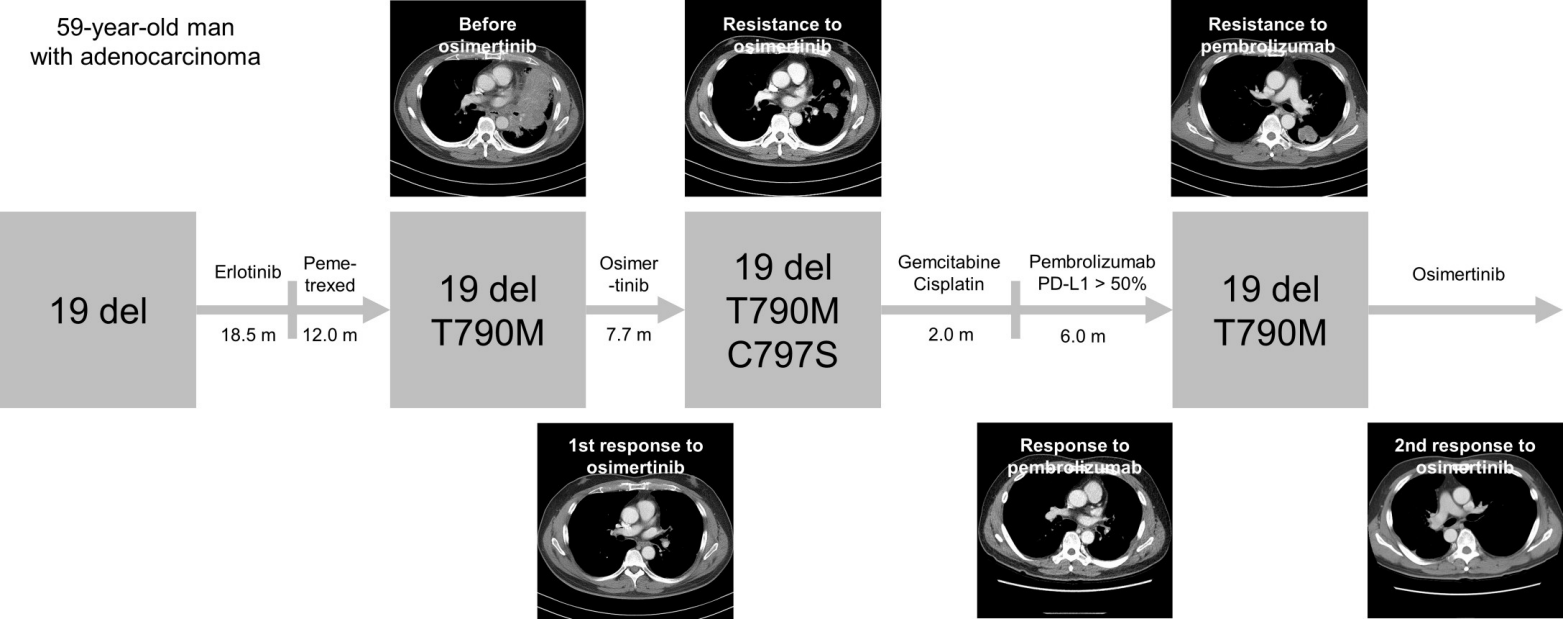
Longitudinal response to treatment in a patient with the C797S mutation.

**Fig 5:**
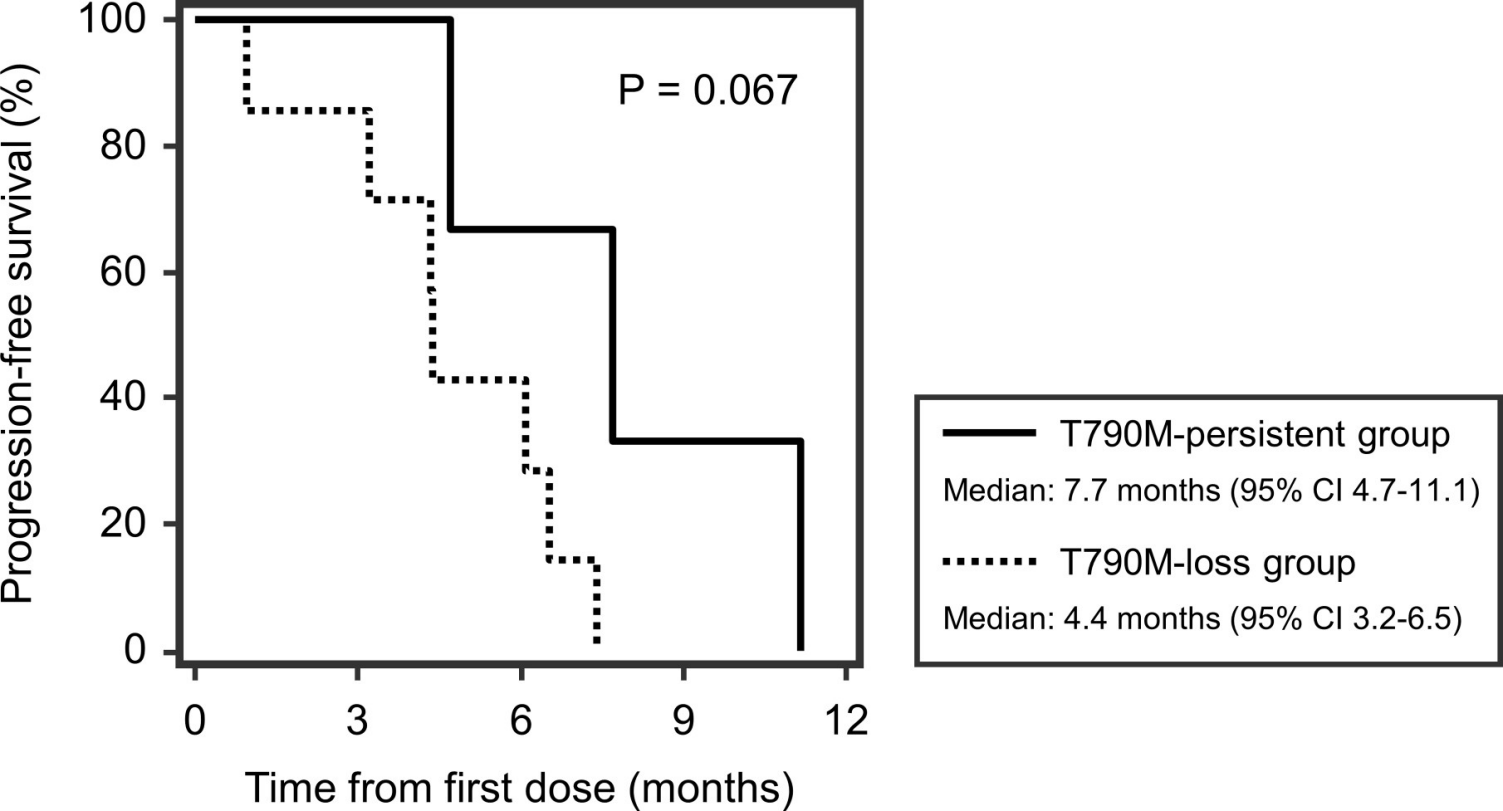
Comparison of progression-free survival between the T790M-persistent group and the T790M-loss group. Abbreviation: CI, confidence interval.

Fourteen patients experienced disease progression during treatment with osimertinib, of which 10 (71.4%) underwent repeat biopsy. The resistance profile of osimertinib is summarized in Table 3. The T790M mutation became undetectable in 7 (70.0%) of the 10 patients who underwent re-biopsy (T790M-loss group), whereas it persisted in 3 (30.0%) patients (T790M-persistent group). In the T790M-loss group, two cases showed transformation to small cell carcinoma that responded well to subsequent chemotherapy with etoposide and cisplatin (patients No. 6 and 8) (Fig 3). Five patients who had “exon 19 deletions plus T790M mutation” before osimertinib treatment showed “exon 19 deletions” only at the time of disease progression. Of two patients with “L858R point mutation plus T790M mutation” before osimertinib treatment, one lost the “T790M mutation” and showed “L858R point mutation” only at the time of disease progression (patient No. 10), and one showed “wild-type conversion” (patient No. 5). In the T790M-persistent group, there was one case of newly developed C797S mutation concurrent with exon 19 deletions and the T790M mutation (patient No. 9) (Fig 4). The patient received two cycles of systemic chemotherapy with gemcitabine and cisplatin and six cycles of pembrolizumab, after which a repeat biopsy showed that the C797S mutation had disappeared. The molecular pathologic analysis of re-biopsied tissue revealed the presence of the T790M mutation and exon 19 deletions, and the patient was retreated with osimertinib, showing a good response to the drug. Comparison of the PFS between the T790M-loss group and the T790M-persistent group showed a trend toward worse PFS in the T790M-loss group (4.4 vs. 7.7 months, P = 0.067) (Fig 5).

**Table 3:** Resistance profile of osimertinib in real-world practice.

Group	Patient no.	Before osimertinib		After resistance to osimertinib		Progression-free survival (months)	
		Histology	Mutation	Histology	Mutation	First EGFR-TKI	Osimertinib
T790M-loss	1	Adeno-carcinoma	T790M E19del.	Adeno-carcinoma	E19del.	13.0 (Afatinib)	1.0
	2	Adeno-carcinoma	T790M E19del.	Adeno-carcinoma	E19del.	10.1 (Gefitinib)	6.1
	4	Adeno-carcinoma	T790M E19del.	Adeno-carcinoma	E19del.	32.5 (Gefitinib)	3.2
	5	Adeno-carcinoma	T790M L858R	Adeno-carcinoma	Wild type	8.2 (Gefitinib)	4.4
	6	Adeno-carcinoma	T790M E19del.	Small cell carcinoma	E19del.	12.0 (Gefitinib)	4.3
	8	Adeno-carcinoma	T790M E19del.	Small cell carcinoma	E19del.	18.0 (Gefitinib)	6.5
	10	Adeno-carcinoma	T790M L858R	Adeno-carcinoma	L858R	14.0 (Gefitinib)	7.4
T790M-persistent	3	Adeno-carcinoma	T790M L858R	Adeno-carcinoma	T790M L858R	6.3 (Gefitinib)	11.1
	7	Adeno-carcinoma	T790M E19del.	Adeno-carcinoma	T790M E19del.	11.8 (Gefitinib)	4.7
	9	Adeno-carcinoma	T790M E19del.	Adeno-carcinoma	T790M E19del. C797S	18.5 (Erlotinib)	7.7

Abbreviation: EGFR-TKI, epidermal growth factor receptor-tyrosine kinase inhibitor.

## Discussion

The efficacy of osimertinib determined in the present study was comparable to that reported previously [21, 24], showing an ORR of 73.9% and a PFS of 7.4 months. Excluding one case of severe pneumonitis leading to discontinuation of the drug, osimertinib showed a manageable toxicity profile with minimal adverse events. The present results indicate that osimertinib is an effective and safe treatment option for T790M mutation-positive NSCLC in real-world practice.

Acquired resistance is an inevitable problem associated with most targeted agents. In the present study, most of the patients who responded well to osimertinib experienced disease progression within 1 year. Because osimertinib is a relatively newly released drug, the number of patients acquiring resistance to the drug is expected to grow rapidly. Therefore, resistance mechanisms need to be elucidated to design strategies for overcoming such resistance. Several mechanisms of resistance to osimertinib have been proposed [28, 29]. The newly developed C797S mutation, which impairs the covalent binding of osimertinib to EGFR, is an important mechanism of resistance to the drug [30, 31]. This mutation affects other EGFR mutant-selective inhibitors such as olmutinib, HKI-272, and WZ4002 [32–35]. In previous work from our group, we confirmed the ineffectiveness of a new third-generation EGFR-TKI, OBX1-012, the efficacy of which is comparable to that of osimertinib for controlling Ba/F3 cells with forced expression of del/T790M/C797S or L858R/T790M/C797S [36].

In the present study, one patient acquired the C797S mutation with persistent sensitizing exon 19 deletion and T790M mutations. However, the C797S mutation became undetectable after two cycles of systemic chemotherapy with gemcitabine and cisplatin followed by six cycles of pembrolizumab, whereas the sensitizing exon 19 deletion and T790M mutation persisted. Retreatment with osimertinib caused tumor regression. To the best of our knowledge, this is the first case of restoration of sensitivity to osimertinib after systemic treatment in a patient who acquired the C797S mutation. High expression levels of programmed cell-death ligand 1 (PD-L1), an important predictive biomarker for immune-checkpoint inhibitors (ICIs) such as pembrolizumab and nivolumab, occur with relatively low frequency in the EGFR mutant group; therefore, ICIs are generally less effective in patients with EGFR mutations [37–40]. However, in the present study, the indicated patient showed high PD-L1 expression, suggesting that the patient would respond well to subsequent treatment with pembrolizumab. In addition, a high tumor mutation burden (TMB) is associated with the response to ICIs [41]; therefore, a high TMB in the indicated patient would suggest the existence of a heterogeneous tumor population. Although the heterogeneity of the tumor population was assessed only for EGFR mutations in the present study, the restoration of sensitivity to osimertinib after systemic treatment may reflect the existence of a heterogeneous tumor population that could be altered in response to different pharmacologic pressures. This case underscores the need to monitor mutational profiles in EGFR mutant lung cancer in parallel with the changes in drug regimens, which may help determine the appropriate subsequent treatment.

Thress et al. evaluated the status of EGFR mutations in 15 osimertinib-treated patients using droplet digital PCR of serial cell-free DNA specimens [30]. These authors reported that the T790M mutation was lost in four cases, whereas it was maintained in the remaining cohort, of which six patients acquired a newly developed C797S mutation together with the persistent T790M mutation. Consistently, we found that the T790M mutation vanished in seven patients including one case of EGFR wild-type conversion. The patients showing loss of the T790M mutation without the C797S mutation may have an active EGFR-independent resistance mechanism such as bypass signal activation, as osimertinib also controls EGFR-sensitizing mutations.

In the present study, the T790M-loss group showed a trend toward shorter PFS than the T790M-persistent group (4.4 vs. 7.7 months, P = 0.067). This is consistent with a previous study by Oxnard et al., in which a worse prognosis was associated with the earlier development of new metastases in the T790M-negative group [42]. These authors indicated that patients without the T790M mutation in the resistant setting may harbor additional alternative mechanisms of resistance such as MET amplification, leading to a worse prognosis. Moreover, a recent study using next generation sequencing (NGS) confirmed that loss of the T790M mutation is associated with a shorter PFS and a shorter time to treatment discontinuation (TTD) and with a range of competing resistance mechanisms, indicating that the resistance to osimertinib in this group may be mediated by the emergence of pre-existing resistant clones [43].

Two patients in the present study exhibited transformation to small cell carcinoma and responded well to the standard chemotherapy with etoposide and cisplatin. Transformation to small cell carcinoma occurs frequently during treatment with first- or second-generation EGFR-TKIs [12, 44]; therefore, such transformation is a common phenomenon for all-generation EGFR-TKIs. In three patients, repeat biopsy led to the design of a therapeutic plan following resistance to osimertinib. This underscores the importance of repeat biopsy at the time of disease progression or later to direct the subsequent therapeutic plan.

In the present study, osimertinib showed a manageable toxicity profile with minimal adverse events except for one case of pneumonitis. However, the detailed toxicity profile differed from that observed in previous clinical trials such as the AURA3 trial [21]. For example, abnormalities in blood cell counts, including leukopenia, neutropenia, anemia, and thrombocytopenia, and in liver function tests were more common in the present study than in the AURA3 trial. On the other hand, diarrhea and skin toxicities were less frequently observed in the present study. Because we aimed to investigate the efficacy, safety, and resistance profile of osimertinib in real-world practice, we did not control for concurrently administered medications such as antibiotics that may affect the blood cell counts and liver function tests. Moreover, the included patients were allowed to take over-the-counter medications such as anti-histamines. These factors could contribute to the differences in the toxicity profile of osimertinib between studies. In addition, literature reports show that GI toxicity such as diarrhea is relatively less common in the Japanese population [45], suggesting the potential effect of ethnic differences on the variation in toxicity profiles.

The present study had several limitations. First, the retrospective design and relatively small sample size limited the conclusions. Second, osimertinib was initiated in different lines. Third, the exclusion of patients enrolled in other clinical trials may be associated with bias, as the medical and performance status of the included patients might be inferior to that of the excluded population. Although the demographic and clinicopathologic data including age, gender, and the proportion of patients with CNS metastases and with exon 19 deletion and L858R mutation in the present study were comparable to those of previous clinical trials [21, 46], our findings should be confirmed in further studies with an improved design. Fourth, the present study used two different methods for the molecular diagnosis of EGFR mutations. However, the two methods, direct sequencing and PNA clamping-based real-time PCR, showed comparable sensitivity in our previous study [27]. Moreover, definition of the molecular pathologic resistance profile of osimertinib, which was one of the main results of this study, was performed solely by PNA clamping-based real-time PCR. Therefore, the use of different methods of molecular diagnosis likely had a minimal effect on the study results. Fifth, because the investigation was limited to mutations in the EGFR gene, other possible resistance mechanisms such as MET amplification were not assessed in the present study. Further studies are required to fully address the mechanisms of resistance of osimertinib.

In conclusion, the present retrospective observational study showed that osimertinib had favorable clinical activity and toxicity profile in real-world practice comparable to those observed in clinical trials. Repeat biopsy after the acquisition of resistance to osimertinib is important to design subsequent treatment strategies.

## Supporting information

S1 Table Safety overview of adverse events by CTCAE 5.0. Data are presented as number (%).
Abbreviations: ALT, alanine aminotransferase; AST, aspartate aminotransferase; CPK, creatinine phosphokinase; CTCAE, Common Terminology Criteria for Adverse Events; WBC, white blood cell.
(DOCX)
