# Supplementary material for: Efficacy, safety, and resistance profile of osimertinib in T790M mutation-positive non-small cell lung cancer in real-world practice
Source: PLoS One. 2019 Jan 9;14(1):e0210225. doi: 10.1371/journal.pone.0210225 (PMC6326493; doi:10.1371/journal.pone.0210225)
Supplement: S1 Table — Data are presented as number (%). Abbreviations: ALT, alanine aminotransferase; AST, aspartate aminotransferase; CPK, creatinine phosphokinase; CTCAE, Common Terminology Criteria for Adverse Events; WBC, white blood cell. (DOCX) [file pone.0210225.s001.docx]

**S1 Table. Safety overview of adverse events by CTCAE 5.0.**

|  | **Any grade** | **Grade 1** | **Grade 2** | **Grade 3** | **Grades 4–5** |
| --- | --- | --- | --- | --- | --- |
| Anemia | 15 (65.2) | 10 (43.5) | 2 (8.7) | 3 (13.0) | 0 (0.0) |
| Decreased platelet count | 10 (43.5) | 7 (30.4) | 2 (8.7) | 1 (4.3) | 0 (0.0) |
| Decreased WBC count | 8 (34.8) | 6 (26.1) | 1 (4.3) | 1 (4.3) | 0 (0.0) |
| Decreased neutrophil count | 14 (60.9) | 10 (43.5) | 2 (8.7) | 2 (8.7) | 0 (0.0) |
| Increased ALT | 10 (43.5) | 9 (39.1) | 1 (4.3) | 0 (0.0) | 0 (0.0) |
| Increased AST | 7 (30.4) | 5 (21.7) | 1 (4.3) | 1 (4.3) | 0 (0.0) |
| Hyponatremia | 4 (17.4) | 4 (17.4) | 0 (0.0) | 0 (0.0) | 0 (0.0) |
| Increased CPK | 1 (4.3) | 1 (4.3) | 0 (0.0) | 0 (0.0) | 0 (0.0) |
| Rash (grouped term) | 2 (8.7) | 2 (8.7) | 0 (0.0) | 0 (0.0) | 0 (0.0) |
| Dry skin | 2 (8.7) | 1 (4.3) | 1 (4.3) | 0 (0.0) | 0 (0.0) |
| Pruritus | 2 (8.7) | 2 (8.7) | 0 (0.0) | 0 (0.0) | 0 (0.0) |
| Paronychia | 4 (17.4) | 3 (13.0) | 1 (4.3) | 0 (0.0) | 0 (0.0) |
| Stomatitis | 1 (4.3) | 1 (4.3) | 0 (0.0) | 0 (0.0) | 0 (0.0) |
| Pharyngitis | 1 (4.3) | 0 (0.0) | 1 (4.3) | 0 (0.0) | 0 (0.0) |
| Nausea | 11 (47.8) | 6 (26.1) | 5 (21.7) | 0 (0.0) | 0 (0.0) |
| Vomiting | 4 (17.4) | 2 (8.7) | 2 (8.7) | 0 (0.0) | 0 (0.0) |
| Diarrhea | 3 (13.0) | 3 (13.0) | 0 (0.0) | 0 (0.0) | 0 (0.0) |
| Constipation | 2 (8.7) | 0 (0.0) | 2 (8.7) | 0 (0.0) | 0 (0.0) |
| Fatigue | 11 (47.8) | 5 (21.7) | 6 (26.1) | 0 (0.0) | 0 (0.0) |
| Pneumonitis | 1 (4.3) | 0 (0.0) | 0 (0.0) | 1 (4.3) | 0 (0.0) |
| Lung infection | 1 (4.3) | 0 (0.0) | 1 (4.3) | 0 (0.0) | 0 (0.0) |
| Cough | 13 (56.9) | 10 (43.5) | 3 (13.0) | 0 (0.0) | 0 (0.0) |
| Dyspnea | 11 (47.8) | 5 (21.7) | 6 (26.1) | 0 (0.0) | 0 (0.0) |
| Pulmonary embolism | 1 (4.3) | 0 (0.0) | 0 (0.0) | 1 (4.3) | 0 (0.0) |
| Headache | 5 (21.7) | 2 (8.7) | 3 (13.0) | 0 (0.0) | 0 (0.0) |
| Back pain | 6 (26.1) | 1 (4.3) | 5 (21.7) | 0 (0.0) | 0 (0.0) |
| Fever | 4 (17.4) | 3 (13.0) | 1 (4.3) | 0 (0.0) | 0 (0.0) |
| Malaise | 2 (8.7) | 2 (8.7) | 0 (0.0) | 0 (0.0) | 0 (0.0) |

Data are presented as number (%).

Abbreviations: ALT, alanine aminotransferase; AST, aspartate aminotransferase; CPK, creatinine phosphokinase; CTCAE, Common Terminology Criteria for Adverse Events; WBC, white blood cell.
